# Large indel detection in region-based phased diploid assemblies from linked-reads

**DOI:** 10.1186/s12864-025-11398-z

**Published:** 2025-03-18

**Authors:** Can Luo, Brock A. Peters, Xin Maizie Zhou

**Affiliations:** 1 Department of Biomedical Engineering, Vanderbilt University, Nashville, 37235 TN USA; 2 Advanced Genomics Technology Lab, Complete Genomics Inc, 2904 Orchard Parkway, San Jose, 95134 CA USA; 3 Department of Computer Science, Vanderbilt University, Nashville, 37235 TN USA

**Keywords:** Structural variants, Phasing, Region-based, Diploid assembly, Linked-reads

## Abstract

**Background:**

Linked-reads improve de novo assembly, haplotype phasing, structural variant (SV) detection, and other applications through highly-multiplexed genome partitioning and barcoding. Whole genome assembly and assembly-based variant detection based on linked-reads often require intensive computation costs and are not suitable for large population studies. Here we propose an efficient pipeline, RegionIndel, a region-based diploid assembly approach to characterize large indel SVs. This pipeline only focuses on target regions (50kb by default) to extract barcoded reads as input and then integrates a haplotyping algorithm and local assembly to generate phased diploid contiguous sequences (contigs). Finally, it detects variants in the contigs through a pairwise contig-to-reference comparison.

**Results:**

We applied RegionIndel on two linked-reads libraries of sample HG002, one using 10x and the other stLFR. HG002 is a well-studied sample and the Genome in a Bottle (GiaB) community provides a gold standard SV set for it. RegionIndel outperformed several assembly and alignment-based SV callers in our benchmark experiments. After assembling all indel SVs, RegionIndel achieved an overall F1 score of 74.8% in deletions and 61.8% in insertions for 10x linked-reads, and 64.3% in deletions and 36.7% in insertions for stLFR linked-reads, respectively. Furthermore, it achieved an overall genotyping accuracy of 83.6% and 80.8% for 10x and stLFR linked-reads, respectively.

**Conclusions:**

RegionIndel can achieve diploid assembly and detect indel SVs in each target region. The phased diploid contigs can further allow us to investigate indel SVs with nearby linked single nucleotide polymorphism (SNPs) and small indels in the same haplotype.

## Background

Structural variants (SVs) represent large genomic variations that encompass at least 50bp. They occur as a result of DNA deletions, insertions, duplications, inversions, or translocations, which result in different combinations of DNA gains, losses, or rearrangements [1]. SVs are related to various rare diseases and cancer, and for this reason, identifying SVs is a significant objective in genomic studies. Despite the importance of detecting SVs, current sequencing technology limitations and the complexity of SVs are making it challenging to make progress in this area.

The success of The Human Genome Project spurred a plethora of downstream analysis approaches in applying whole genome sequencing (WGS) to diverse research questions [2]. Illumina short-read sequencing was the first WGS approach and remains the most popular platform for genome-wide characterizations. Short-read sequencing presents advantages such as low sequencing error and low cost. Numerous methods and tools have been developed to detect genome-wide variants. However, due to the inherent limitations of short reads, its utility is more limited for the accurate detection of SVs, and especially larger ones, spanning 500bp or more in human genomes [3–5]. Additionally, phasing (assigning genetic variants to their homologous chromosome of origin) is important for genome interpretation and functional analysis of allelic activity, but it is also beyond the ability of short reads sequencing.

To overcome the limitation of traditional short reads sequencing, long reads sequencing technologies, including Pacific Biosciences (PacBio) and Oxford Nanopore (ONT) Technologies [6, 7], have been developed. Long reads sequencing provides a novel solution by fragmenting the DNA molecule into longer pieces, typically thousands of base pairs for PacBio, and as long as hundreds of thousands for ONT. Such technology naturally resolves the problem of calling large SVs, but this achievement is diminished by its stringent input requirements, high error rate at the base level, and high cost [4, 8]. The latest technique can now generate long reads with high accuracy (>99%, such as PacBio HiFi) via circular consensus sequencing (CCS), however, it requires at least three subreads from the same molecule to achieve such accuracy [9]. Thus, long reads sequencing is substantially limited in sequencing a large population of samples for population studies. Compared to long reads sequencing, a third technology, linked-read sequencing presents a viable alternative since it inherits all advantages of short reads sequencing while retaining long-range information.

Linked-reads technology was originally developed by Complete Genomics and popularized by 10x Genomics, but the availability of the 10x linked-reads products was discontinued recently, apparently due to a patent dispute [10, 11]. Several other companies have offered alternatives in this promising technology arena, such as single tube long fragment reads (stLFR) by MGI [12], and TELL-Seq by Universal Sequencing Technology [13]. High-quality 10x linked-reads data were widely used for half a decade and demonstrated their advantage in phasing, SV calling, and diploid assembly [14–21]. The original, 10x linked-read data were produced through the following process: long DNA fragments are first diluted into ${10^5}{\sim }{10^6}$ microfluidic droplets. Within each droplet, a randomly primed amplification then produces many identically barcoded short fragments (linked-reads) [22]. In the case of stLFR from MGI, linked-reads are derived by adding the same barcode sequence to sub-fragments of the original long DNA molecule (DNA co-barcoding). To achieve this efficiently, stLFR uses the surface of microbeads to create millions of miniaturized barcoding reactions in a single tube [12]. Universal Sequencing Technology performs a process similar to stLFR by developing single-tube Transposase Enzyme Linked Long-read Sequencing (TELL-Seq) technology. The key process of producing TELL-Seq linked reads begins with millions of clonally barcoded beads in a PCR tube that are used to uniquely barcode long DNA molecules in an open bulk reaction without dilution and compartmentation [13]. In summary, even though the biological processes are different in different kinds of linked-reads sequencing technologies, the critical, shared concept is that each group of reads that share the same barcode is drawn from the same fragment (or several different distant fragments). By utilizing barcode information, we can reconstruct long-range information from barcoded reads.

By fully taking advantage of 10x linked-reads, the native software tool Supernova introduced by 10x Genomics generated diploid, phased assemblies through a whole genome de novo assembly approach [23]. It is then straightforward to perform reference-to-contig pairwise comparisons to detect SVs. Several state-of-the-art methods and tools such as LongRanger [24], GROC-SVs [16], NABIR [14], Novel-X [15], and LinkedSV [25] have been developed to characterize SVs for linked-reads through alignment-based approaches. Compared to alignment-based approaches, de novo assembly-based approaches are less influenced by the reference and enable more comprehensive and unbiased SV detection because of their inherent reference-free strategy. Recently, Aquila and Aquila_stLFR were introduced to perform diploid personal genome assembly and genome-wide structural variant detection for 10x and stLFR linked-reads, respectively [18, 26]. They use a hybrid method to take advantage of the high-quality reference genome and local de novo assembly, to generate phased diploid contigs at the whole genome scale. Compared to the whole genome de novo assembly approach, this hybrid approach further improves phased assembly quality and SV calls in linked-reads.

Even though whole genome assembly is a promising strategy to investigate and characterize individual personal genomes, it is computationally prohibitive for large population studies due to high demands for memory and computing time. Therefore, we were motivated to introduce RegionIndel, an indel SV calling approach through region-based phased diploid assemblies. This pipeline has two prominent advantages: the region-based strategy requires less time and computation resources than the whole genome, making it more likely to be used for large population studies; the assembled phased contiguous sequences (contigs) still allow us to investigate nearby linked variants for each SV from both haplotypes. In this paper, we applied RegionIndel on two HG002 linked-reads libraries, one generated with 10x and the other with stLFR linked-reads. We evaluated SV calls ($\ge$50bp) relative to the Genome in a Bottle (GiaB) gold standard through Truvari [27], an SV benchmarking tool. The results showed RegionIndel achieved an overall F1 score of 74.8% in deletions for 10x, and 64.3% in deletions for stLFR linked-reads, respectively. Overall, the accuracy of detection for insertions was lower than for deletions. RegionIndel reached an overall F1 score of 61.8% for 10x, and 36.7% for stLFR linked-reads, respectively. Our benchmark experiments also demonstrated that RegionIndel outperformed two whole genome assembly-based SV callers (Aquila and Aquila_stLFR) and two alignment-based SV callers (Novel-X and LinkedSV). Moreover, the phased diploid contigs from RegionIndel could allow us to investigate the nearby linked single nucleotide polymorphisms (SNPs) for each indel SV from different parental haplotypes.

**Fig. 1:**
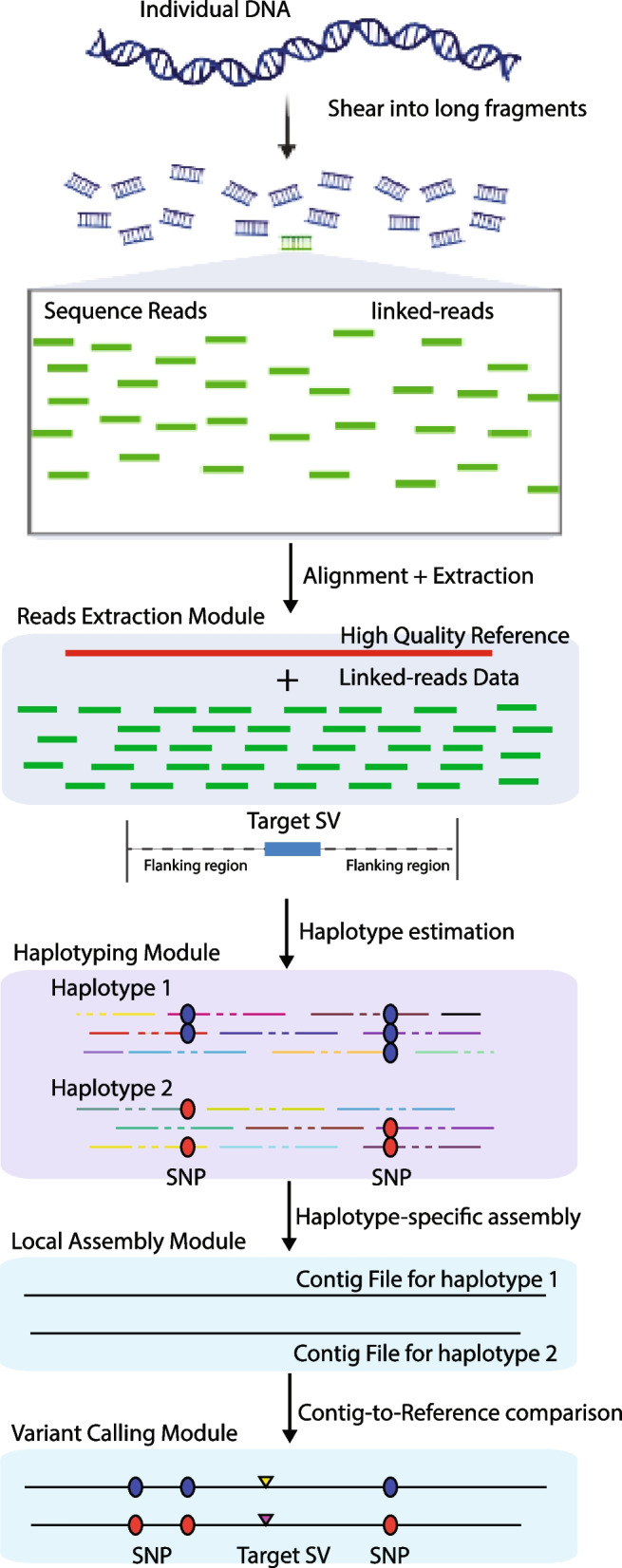
Schematic diagram of the RegionIndel pipeline. Input data are the high-quality reference genome and barcoded reads, each with a barcode (not shown). The reads extraction module extracts barcoded reads aligning to the region of interest. The reads with the same barcode (read cloud) form the virtual long fragment molecule. The haplotyping module partitions molecules into different parental haplotypes, and then the corresponding barcoded reads will be partitioned into different haplotypes. The local assembly module takes barcoded reads and performs de novo local assembly independently for each haplotype through SPAdes. Finally, the variant calling module integrates paftools to perform a contig-to-reference comparison to detect all variants

## Methods

The whole pipeline of RegionIndel is illustrated in Fig. 1. The input files for RegionIndel are a BAM file and a VCF file. To generate the BAM file, we can align the raw reads FASTQ file to the human reference through LongRanger [24], EMA [28], or BWA [29]. We generate the VCF file through the variant caller FreeBayes [30]. The VCF file includes all the heterozygous SNPs, which RegionIndel utilizes to annotate long DNA molecules and perform haplotyping.

In the reads extraction module, RegionIndel only extracts barcoded reads that align to the region of interest from the BAM file. To guarantee all correct barcoded reads land in the region of interest, the algorithm needs first to solve the barcode deconvolution problem. RegionIndel seeks to differentiate reads with the same barcode into different groups (also called read clouds) to guarantee that reads in the same read cloud are drawn from the same long DNA fragment molecule. Once this is solved, RegionIndel could simply pull out all read clouds aligned to the target region and reconstruct each virtual DNA molecule based on each read cloud. To overcome the barcode deconvolution problem, we use an alignment-based method to apply an empirical boundary threshold (e.g. 50kb for 10x linked-reads, 20kb for stLFR linked-reads) to differentiate read clouds with the same barcode. For instance, if the distance between two successive reads with the same barcode is larger than the boundary threshold, they will be assumed to be drawn from two different fragment molecules. After reconstructing all virtual fragment molecules *L* (read clouds) in the region of interest, we annotate each molecule by heterozygous SNPs (“0” for reference allele and “1” for alternate allele) through the barcoded reads assigned to the molecule. For instance, if a molecule is annotated by “chr1, 2345677:0, 3677888:1, 8900543:1”, it implies that this molecule covers a reference allele at locus 2345677 of chromosome 1, an alternate allele at locus 3677888, and an alternate allele at locus 8900543.

The haplotyping module relies on all pairs of annotated heterozygous SNPs of every molecule and a recursive clustering algorithm that we developed recently [18] to partition long fragment molecules *L* into either maternal or paternal haplotypes within each phase block. The essential underlying idea is that the maternal and paternal haplotypes always represent complementary alleles for each heterozygous variant (e.g. if the maternal haplotype represents “01001” for five heterozygous SNPs, then the paternal haplotype represents “10110”). Thus, it can also partition all barcoded reads belonging to long molecules (read clouds) into two parental haplotypes. Ideally, there is only one phase block for the region of interest, and RegionIndel denotes $R_{HP1}$ as barcoded reads partitioning into haplotype 1 (HP1), and $R_{HP2}$ as barcoded reads partitioning into haplotype 2 (HP2) within each phase block.

The local assembly module uses the short reads assembler SPAdes [31] to assemble $R_{HP1}$ and $R_{HP2}$, respectively. Extracted reads $R_{HP1}$ and $R_{HP2}$ within the region of interest will be expected to receive enough coverage and can be assembled into contiguous sequences (contigs) for each haplotype within the phase block. The output of this module is haplotype-resolved contigs for each haplotype.

The last, variant calling module integrates minimap2 [32] to perform a pairwise contig-to-reference comparison for each set of haplotype-resolved contigs and generate a paf file which contains all types of variants for each haplotype within a certain phase block. RegionIndel then employs paftools [32] to extract variants (including SNPs, small and large indels) from each paf file, to produce a set of variants. Once a set of variants for each haplotype within each phase block is generated, lastly, RegionIndel merges and genotypes the variants from two sets (haplotypes). Specifically, it first sorts the two sets of variants by position independently, then compares variants from both sets. The variants that are shared by both sets are genotyped as 1|1, and the variants that are only included in set 1 are genotyped as 1|0, otherwise 0|1 with a certain phase block. Furthermore, due to the artifact of assembly, there might be overlapping contigs even within one haplotype, we thus use a chaining algorithm to reduce the redundancy. The chaining algorithm checks any pair of variants of the same type that are within a distance threshold (500bp by default) and compares their similarity (sequence and size similarity) to determine if they need to be merged into one variant or not. Moreover, alignment-based methods like FreeBayes often achieve near-perfect SNP calling, RegionIndel thus relies on SNPs from FreeBayes to further refine the contig-based SNPs. Finally, RegionIndel outputs all kinds of heterozygous and homozygous variants in a phased VCF file.

## Results

We applied RegionIndel to two different linked-reads libraries generated with 10x and stLFR for sample HG002 to perform region-based diploid assembly and SV calling [17, 33]. There are several important parameters that describe linked-reads libraries, such as raw coverage (*C*), the average coverage of short reads per fragment ($C_R$), average physical coverage of the genome by long DNA fragments ($C_F$), and mean unweighted DNA fragment length ($\mu _{FL}$). Parameters of those two libraries are listed here for the stLFR library: *C*=48X, $C_F$=238X, $C_R$=0.20X, $\mu _{FL}$=30.1kb; and for the 10x library: *C*=93X, $C_F$=760X, $C_R$=0.12X, $\mu _{FL}$=44.8kb.

We used the Genome in a Bottle (GiaB) benchmark v0.6 as the gold standard in this study [34]. This benchmark is specific to HG002 and the hg19 reference sequence, with 9397 SVs $\ge$50bp in high-confidence regions. We calculated the recall, precision, and F1 score of the RegionIndel calls by assuming all these GiaB calls were correct. We used the open-source tool Truvari [27] to compare the RegionIndel calls to the benchmark. The parameters we used to run Truvari: *p=0.1, P=0.1, r=200, O=0*.

As mentioned, RegionIndel utilizes a region-based diploid assembly approach to detect SVs. To generate the input files and evaluate all SVs of high-confidence regions from the benchmark, we extract barcoded reads from each high-confidence SV region by selecting a left and right flanking region (25kb by default) around the breakpoints of each SV.

**Fig. 2:**
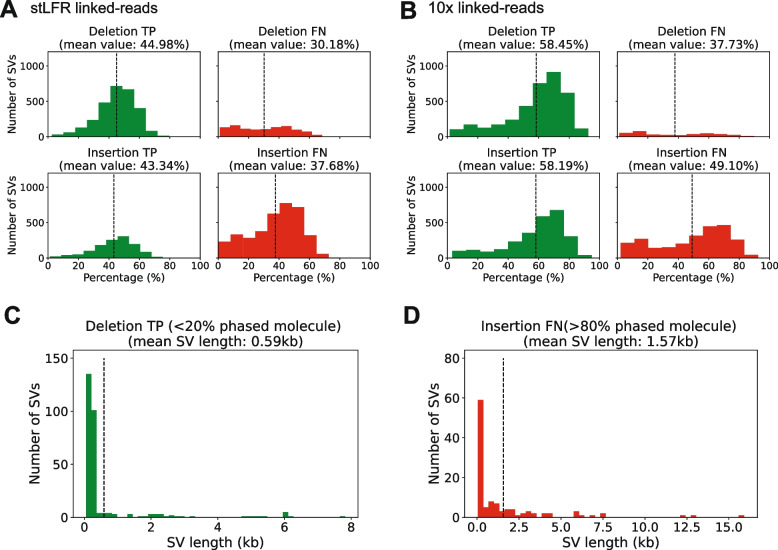
The distribution plot of the number of SVs as a function of the percentage of phased molecules or SV length. **A** The distribution plot as a function of the percentage of phased molecules in stLFR linked-reads for true positive (TP) and false negative (FN) deletions (top panels) and TP and FN insertions (bottom panels). **B** The distribution plot as a function of the percentage of phased molecules in 10x linked-reads for TP and FN deletions (top panels) and TP and FN insertions (bottom panels). Each vertical line represents the average percentage of phased molecules. **C** The distribution plot as a function of SV length for TP deletions (<20% phased molecules) **D** The distribution plot as a function of SV length for FN insertions (>80% phased molecules)

### Effect of the percentage of phased molecules on indel SV calls

Partitioning molecules (phasing) is an important process since it divides barcoded reads into two parental haplotypes for assembly. Phasing is applicable for a molecule if it contains at least one heterozygous SNP. Since RegionIndel only partitions barcoded reads belonging to the phased molecules for haplotype-specific assembly, it is not difficult to hypothesize that the more molecules are available to be partitioned into different haplotypes, the more barcoded reads can be used for assembly, resulting in more accurate SV calling. To verify this hypothesis, for each target SV region, we calculated the total percentage of the phased molecules used for later assembly. We also divided the SV calls from RegionIndel into two categories after comparing them to the GiaB benchmarks: True Positive (TP) and False Negative (FN). TP means RegionIndel called the high-confidence SV as identified in the benchmark; FN means that the SV was missed by RegionIndel. We generated the distribution plot of the number of SVs as a function of the percentage of phased molecules, for all different categories for both stLFR and 10x linked-reads libraries in Fig. 2.

To investigate if there is any difference between different types of SVs, we separated TPs and FNs into deletions and insertions. For stLFR data, we observed that for deletions, the average percentage of phased molecules in TPs was 44.98%, while the average percentage of phased molecules in FNs was 30.18%, which was 14.80% less. For insertions, the average percentage of phased molecules in TPs was 43.34%, while the average percentage of phased molecules in FNs was 37.68%, which was 5.66% less (Fig. 2A). Similar results were observed in the 10x results. For deletions, the average percentage of phased molecules in TPs was 58.45%, while the average percentage of phased molecules in FNs was 37.73%, which was 20.72% less. For insertions, the average percentage of phased molecules in TPs was 58.19%, while the average percentage of phased molecules in FNs was 49.10%, which was 9.09% less (Fig. 2B).

All the above results suggest that the higher the percentage of phased molecules, the more accurate the SV detection. Notably, TP deletions can sometimes be detected even with a very low percentage (<20%) of phased molecules, but these cases are rare. These might occur in unique and non-repetitive mapping regions or when the size of deletions is relatively small (Fig. 2C). Conversely, we see some regions with high percentages of phased molecules (>80%) also return FN results in insertion calling and the size of insertions is relatively large (Fig. 2D). The cause of this error might be that reads that contain the novel sequence are absent in the input BAM file because they are not mapped onto the reference genome.

### Evaluation of indel SV calls from region-based diploid assembly

To evaluate the performance of RegionIndel, we divided SVs into three groups according to their sizes: 50bp-1kb, 1kb-10kb, and >10kb (Table 1). For the range of 50bp - 1kb deletions, there were 3605 benchmark deletions and RegionIndel achieved an F1 score of 64.6% for stLFR and 73.4% for 10x, respectively. In the 1kb-10kb size range, there were 481 benchmark deletions and RegionIndel achieved an F1 score of 65.1% for stLFR and 88.1% for 10x, respectively. In the >10kb size range, there were 29 benchmark deletions and RegionIndel achieved an F1 score of 19.5% for stLFR and 52.5% for 10x, respectively.

Because RegionIndel is a diploid assembly-based SV calling approach, we can further evaluate the genotyping results of SVs. RegionIndel showed an overall 82.9% and 87.8% genotyping accuracy for deletions in stLFR and 10x, respectively.

**Table 1:** Evaluation of deletions ($\ge$50bp) in HG002 by RegionIndel, Aquila, Aquila_stLFR, LinkedSV, and Novel-X (chrX and chrY were excluded in the evaluation). Abbreviations: true positives (TP), false positives (FP), false negatives (FN), and genotype (GT)

Deletions		stLFR				10x			
		RegionIndel	Aquila_stLFR	LinkedSV	Novel-X	RegionIndel	Aquila	LinkedSV	Novel-X
50-1k	benchmark	3605							
	TP	2563	2963	8	NaN	3210	3318	2013	NaN
	TP_GT	2116	2547	NaN	NaN	2796	3001	NaN	NaN
	FP	1773	8484	0	NaN	1927	6439	215	NaN
	FN	1042	642	3597	NaN	395	287	1592	NaN
	Recall	71.1%	82.2%	0.2%	NaN	89.0%	92.0%	55.8%	NaN
	Precision	59.1%	25.9%	100.0%	NaN	62.5%	34.0%	90.4%	NaN
	F1	64.6%	39.4%	0.4%	NaN	73.4%	49.7%	69.0%	NaN
	GT_accuracy	82.6%	86.0%	NaN	NaN	87.1%	90.4%	NaN	NaN
1k-10k	benchmark	481							
	TP	296	317	92	NaN	419	365	361	NaN
	TP_GT	252	293	NaN	NaN	390	339	NaN	NaN
	FP	133	285	20	NaN	51	90	54	NaN
	FN	185	164	389	NaN	62	116	120	NaN
	Recall	61.5%	65.9%	19.1%	NaN	87.1%	75.9%	75.1%	NaN
	Precision	69.0%	52.7%	82.1%	NaN	89.1%	80.2%	87.0%	NaN
	F1	65.1%	58.5%	31.0%	NaN	88.1%	78.0%	80.6%	NaN
	GT_accuracy	85.1%	92.4%	NaN	NaN	93.1%	92.9%	NaN	NaN
>10k	benchmark	29							
	TP	8	6	27	NaN	16	7	29	NaN
	TP_GT	8	6	NaN	NaN	16	7	NaN	NaN
	FP	45	99	3	NaN	16	49	25	NaN
	FN	21	23	2	NaN	13	22	0	NaN
	Recall	27.6%	20.7%	93.1%	NaN	55.2%	24.1%	100.0%	NaN
	Precision	15.1%	5.7%	90.0%	NaN	50.0%	12.5%	53.7%	NaN
	F1	19.5%	9.0%	91.5%	NaN	52.5%	16.5%	69.9%	NaN
	GT_accuracy	100.0%	100.0%	NaN	NaN	100.0%	100.0%	NaN	NaN
$\ge$50bp (overall)	benchmark	4116							
	TP	2871	3286	142	NaN	3648	3694	2408	NaN
	TP_GT	2380	2846	NaN	NaN	3204	3350	NaN	NaN
	FP	1947	8868	8	NaN	1990	6571	289	NaN
	FN	1245	830	3974	NaN	468	422	1708	NaN
	Recall	69.8%	79.8%	3.4%	NaN	88.6%	89.7%	58.5%	NaN
	Precision	59.6%	27.0%	94.7%	NaN	64.7%	36.0%	89.3%	NaN
	F1	64.3%	40.4%	6.7%	NaN	74.8%	51.4%	70.7%	NaN
	GT_accuracy	82.9%	86.6%	NaN	NaN	87.8%	90.7%	NaN	NaN

**Table 2:** Evaluation of insertions ($\ge$50bp) in HG002 by RegionIndel, Aquila, Aquila_stLFR, LinkedSV, and Novel-X (chrX and chrY were excluded in the evaluation). Abbreviations: true positives (TP), false positives (FP), false negatives (FN), and genotype (GT)

Insertions		stLFR				10x			
		RegionIndel	Aquila_stLFR	LinkedSV	Novel-X	RegionIndel	Aquila	LinkedSV	Novel-X
50-1k	benchmark	4561							
	TP	1199	1158	NaN	182	2522	2335	NaN	273
	TP_GT	913	823	NaN	NaN	1964	1653	NaN	NaN
	FP	80	211	NaN	19	434	2837	NaN	1003
	FN	3362	3403	NaN	4379	2039	2226	NaN	4288
	Recall	26.3%	25.4%	NaN	4.0%	55.3%	51.2%	NaN	6.0%
	Precision	93.7%	84.6%	NaN	90.5%	85.3%	45.1%	NaN	21.4%
	F1	41.1%	39.1%	NaN	7.6%	67.1%	48.0%	NaN	9.4%
	GT_accuracy	76.1%	71.1%	NaN	NaN	77.9%	70.8%	NaN	NaN
1k-10k	benchmark	696							
	TP	5	2	NaN	15	32	17	NaN	46
	TP_GT	1	1	NaN	NaN	14	1	NaN	NaN
	FP	6	4	NaN	1	6	6	NaN	11
	FN	691	694	NaN	681	664	679	NaN	650
	Recall	0.7%	0.3%	NaN	2.2%	4.6%	2.4%	NaN	6.6%
	Precision	45.5%	33.3%	NaN	93.8%	84.2%	73.9%	NaN	80.7%
	F1	1.4%	0.6%	NaN	4.2%	8.7%	4.7%	NaN	12.2%
	GT_accuracy	20.0%	50.0%	NaN	NaN	43.8%	5.9%	NaN	NaN
$\ge$50bp (overall)	benchmark	5281							
	TP	1204	1160	NaN	197	2554	2354	NaN	322
	TP_GT	914	824	NaN	NaN	1978	1654	NaN	NaN
	FP	81	215	NaN	16	434	2835	NaN	991
	FN	4077	4121	NaN	5084	2727	2927	NaN	4959
	Recall	22.8%	22.0%	NaN	3.7%	48.4%	44.6%	NaN	6.1%
	Precision	93.7%	84.4%	NaN	92.5%	85.5%	45.4%	NaN	24.5%
	F1	36.7%	34.9%	NaN	7.2%	61.8%	45.0%	NaN	9.8%
	GT_accuracy	75.9%	71.0%	NaN	NaN	77.4%	70.3%	NaN	NaN

We further evaluated the performance of RegionIndel on insertion calls (Table 2). Insertions are difficult to detect because unmapped paired reads are absent in the input BAM file and cannot be used for assembling novel sequences. In the 50bp-1kb size range, there were 4561 benchmark insertions and RegionIndel achieved an F1 score of 41.1% for stLFR and 67.1% for 10x, respectively. In the 1kb-10kb size range, there were 696 benchmark insertions and RegionIndel achieved an F1 score of 1.4% for stLFR and 8.7% for 10x, respectively. RegionIndel showed an overall 75.9% and 77.4% genotyping accuracy for insertions in stLFR and 10x, respectively. In general, 10x data had a higher accuracy than stLFR in terms of indel SV calling. The possible reasons are the total coverage of stLFR is much lower than 10x data, and stLFR uses paired 100bp reads instead of 150bp.

Overall, for SV sizes $\ge$50bp, RegionIndel achieved a relatively higher recall but lower precision for deletions (69.8% recall, 59.6% precision and 64.3% F1 for stLFR; 88.6% recall, 64.7% precision and 74.8% F1 for 10x in Table 1), and a relatively lower recall but higher precision for insertions (22.8% recall, 93.7% precision and 36.7% F1 for stLFR; 48.4% recall, 85.5% precision and 61.8% F1 for 10x in Table 2). We compared RegionIndel with two whole genome assembly-based SV callers (Aquila and Aquila_stLFR) and two alignment-based SV callers (Novel-X and LinkedSV), which were designed specifically for linked-reads. For stLFR and 10x data (Table 1), Aquila_stLFR and Aquila achieved an overall F1 score of 40.4% and 51.4% in deletions, and 34.9% and 45.0% in insertions, respectively. RegionIndel outperformed both Aquila_stLFR and Aquila in F1 scores by 1.8% - 23.9%. Compared to assembly-based methods, the performance of alignment-based methods is inferior, especially in insertions (Table 2). LinkedSV reached an overall F1 score of 6.7% and 70.7% in deletions for stLFR and 10x, but could not call any insertions in the GIAB gold standard. Novel-X, proposed to detect insertions in linked-reads, reached an overall F1 score of 7.2% and 9.8% in insertions for stLFR and 10x, respectively. Notably, LinkedSV accomplished a high F1 score in deletions for 10x due to low recall but high precision (58.5% recall and 89.3% precision).

### Impact of length of the flanking region on indel SV calling

RegionIndel is a region-based approach, and the target region by default is defined as the left and right 25kb flanking regions around the breakpoints of each target SV. To investigate the impact of different sizes of flanking regions on the result of SV calling, we deployed RegionIndel to call all benchmark SVs of chromosome 21 using the 10x linked-reads dataset with flanking regions of 10kb, 25kb, 50kb, and 100kb. The SV evaluation results for deletions and insertions are demonstrated in Tables 3 and 4, respectively.

We divided the benchmark SVs into two different size ranges: 50bp-1kb and 1kb-10kb. In the 50bp-1kb size range of deletions, there were 58 benchmark SVs and RegionIndel achieved an F1 score of 67.6%, 64.0%, 54.7%, and 48.5% when the flanking region lengths were 10kb, 25kb, 50kb, and 100kb, respectively. In the 1kb-10kb size range, there were 7 benchmark SVs and RegionIndel achieved a F1 score of 100%, 93.3%, 93.3%, and 93.3% when the flanking region lengths were 10kb, 25kb, 50kb, and 100kb, respectively.

With respect to insertions, in the 50bp-1kb size range, there were 95 benchmark SVs and RegionIndel achieved a F1 score of 50.4%, 59.2%, 63.9%, and 62.4% when the flanking region lengths were 10kb, 25kb, 50kb, and 100kb, respectively. In the 1k-10k size range, there were 16 benchmark SVs and RegionIndel achieved a F1 score of 11.8% for different flanking region lengths.

The impact of the length of the flanking region on SV calls based on stLFR linked-reads produced similar results (Additional file 1: Tables S1-2). Overall, in both deletions and insertions, we observed an increasing trend in recall, but a decreasing trend in precision when the flanking region became larger. Larger flanking regions generally produced higher F1 scores for insertion calling, but lower F1 scores for deletion calling. The underlying reason could be that more barcoded reads got involved in reconstructing molecules and haplotype-specific assembly to detect more SVs, however, more false positives are also introduced, especially for deletions. Users can adjust this flanking region parameter based on their needs to study SVs and linked small variants at target regions.

**Table 3:** Evaluation of deletions ($\ge$50bp) on chromosome 21 in HG002 from 10x linked-reads by different lengths of flanking regions in RegionIndel

Deletions		flanking region (10kb)	flanking region (25kb)	flanking region (50kb)	flanking region (100kb)
50-1k	benchmark	58			
	TP	50	55	55	56
	TP_GT	45	48	52	51
	FP	40	59	88	117
	FN	8	3	3	2
	Recall	86.2%	94.8%	94.8%	96.6%
	Precision	55.6%	48.2%	38.5%	32.4%
	F1	67.6%	64.0%	54.7%	48.5%
	GT_accuracy	90.0%	87.3%	94.6%	91.1%
1k-10k	benchmark	7			
	TP	7	7	7	7
	TP_GT	7	7	7	7
	FP	0	1	1	1
	FN	0	0	0	0
	Recall	100.0%	100.0%	100.0%	100.0%
	Precision	100.0%	87.5%	87.5%	87.5%
	F1	100.0%	93.3%	93.3%	93.3%
	GT_accuracy	100.0%	100.0%	100.0%	100.0%

**Table 4:** Evaluation of insertions ($\ge$50bp) on chromosome 21 in HG002 from 10x linked-reads by different lengths of flanking regions in RegionIndel

Insertions		flanking region (10kb)	flanking region (25kb)	flanking region (50kb)	flanking region (100kb)
50-1k	benchmark	95			
	TP	34	45	53	53
	TP_GT	27	37	40	38
	FP	6	12	18	22
	FN	61	50	42	42
	Recall	35.8%	47.4%	55.8%	55.8%
	Precision	85.0%	78.9%	74.6%	70.7%
	F1	50.4%	59.2%	63.9%	62.4%
	GT_accuracy	79.4%	82.2%	75.5%	71.7%
1k-10k	benchmark	16			
	TP	1	1	1	1
	TP_GT	0	0	0	0
	FP	0	0	0	0
	FN	15	15	15	15
	Recall	6.2%	6.2%	6.2%	6.2%
	Precision	100.0%	100.0%	100.0%	100.0%
	F1	11.8%	11.8%	11.8%	11.8%
	GT_accuracy	0.0%	0.0%	0.0%	0.0%

### Examples of assembled phased diploid sequences and SV calls from RegionIndel

**Fig. 3:**
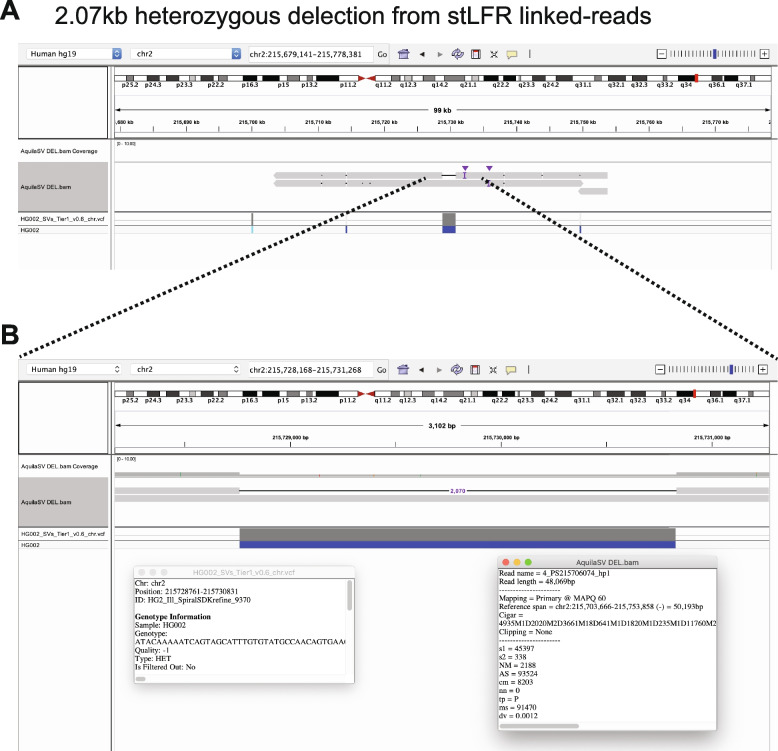
One example of 2.07kb heterozygous deletion from the stLFR linked-reads data by RegionIndel. **A** The IGV displays assembled diploid contigs from AquiaSV. It includes tracks for the contig BAM file from RegionIndel and the GiaB benchmark VCF file. The contigs also show that there are several variants in the nearby region. **B** It is a zoom-in view of the target SV in the center. The left inset box has the description of this benchmark SV, and the right inset box has the description of the contig information generated by RegionIndel. It indicates that this deletion is identified from the contig of haplotype1 and the length of this contig is 48.069kb. The gold standard supports this heterozygous deletion

**Fig. 4:**
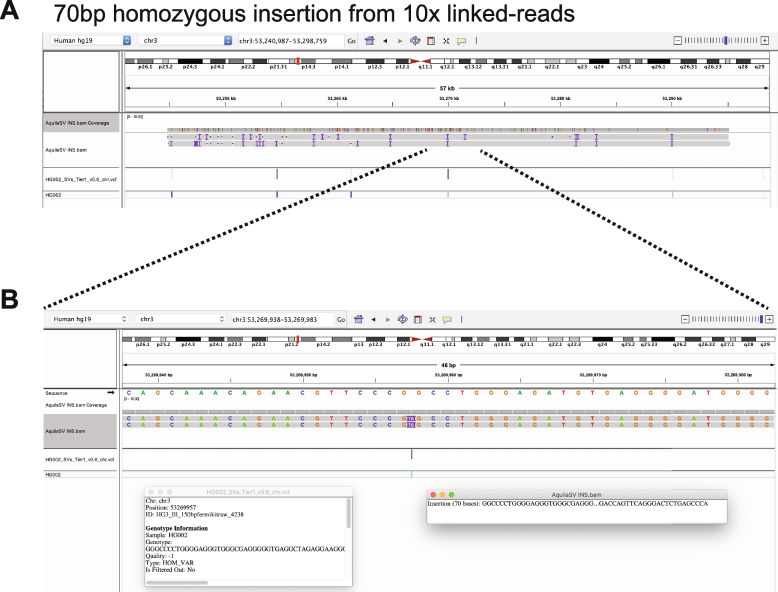
One example of 70bp homozygous insertion from the 10x linked-reads data by RegionIndel. **A** The IGV displays assembled diploid contigs from AquiaSV. It includes tracks for the contig BAM file from RegionIndel and the GiaB benchmark VCF file. The contigs also show there are several variants in the nearby region. **B** It is a zoom-in view of the target SV in the center. The left inset box has the description of this benchmark SV, and the right inset box has the description of the contig information generated by RegionIndel. The gold standard supports this homozygous insertion. The contigs indicate this 70bp insertion is identified by contigs from both haplotypes

To demonstrate the final product of RegionIndel and its functionality, we applied the Integrative Genomics Viewer (IGV) [35] to show successful SV calls. The first SV example in Fig. 3 was a 2.07kb deletion from stLFR linked-reads by RegionIndel. The gold standard suggested that there should be a 2.07kb heterozygous deletion. In the diploid contigs produced by RegionIndel, we could clearly see a deletion in only one of the haploid contigs (the top one). The second example in Fig. 4 was a 70bp insertion from 10x linked-reads. The gold standard suggested that there should be a 70bp homozygous insertion. In the diploid contigs produced by RegionIndel, we could see the two haploid contigs indicate a 70bp insertion in the central region, consistent with the gold standard. We also demonstrated a 5.75kb homozygous deletion from 10x linked-reads and 109bp heterozygous insertion from stFLR linked-reads in Additional file 1: Fig. S1-2.

In addition to the indel SVs displayed in the assembled haplotype-resolved contigs, RegionIndel also identified other variants such as SNPs, small indels, or large indel SVs in the flanking regions, which were found in all example SVs. The phased diploid contigs provided haplotyping results for all variants in each target region.

Finally, we considered memory demands and performance. To assemble one target SV in the region of interest, RegionIndel needed an average of 25 GB of memory and took 10 minutes to finish. In a typical example, RegionIndel finished running at a CPU time of 10min 19s for a target SV located at chromosome 1, position 9370132 (the target SV is from the HG002 Giab benchmark).

## Discussion

We introduce a region-based diploid assembly approach to call SVs in an efficient manner. The products of RegionIndel further allow us to use the actual sequences to phase SV with nearby variants in the flanking regions. RegionIndel is a tool suitable for large population studies when researchers are interested in specific SVs or regions.

To assemble haplotype-resolved contigs in both haplotypes, RegionIndel needs to partition all long molecules (and the corresponding barcoded reads) into two parental haplotypes for local assembly. However, some molecules are not partitioned for later assembly usage if they are too short to cover heterozygous variants or the molecules are involved in the regions without heterozygosity. As shown in the results section, there were still around 50% unphased molecules on average after the haplotyping module. By comparing the results of true positive and false negative SV calls, we could see a big difference in the average percentage of phased molecules and conclude that false negative SVs are more likely to be caused by the lower percentage of phased molecules than true positive SVs. A further step in future studies will be to develop an algorithm to partition the rest of the molecules (read clouds) for local assembly and enhance the accuracy of SV calling. Another issue of RegionIndel is the lower accuracy of insertion SV calling, compared to deletion SVs. One major reason is the absence of unmapped paired reads (both read1 and read2 are not mapped in the reference) in the input BAM file. Another reason is that unphased molecules are not used for later assembly which is mentioned above. Improving insertion SV detection will also be a future step.

## Conclusions

RegionIndel applies a region-based diploid assembly approach to detect SVs in a region of interest for 10x and stLFR linked-reads. It achieves diploid assembly in each target region, and phased contigs further allow us to investigate SVs with nearby linked SNPs, small Indels, and SVs in the same haplotype. RegionIndel provides us with an efficient way to investigate SVs in a potentially large population.

## Supplementary information

**Additional file 1.** Evaluation of deletions ($\ge$50bp) on chromosome 21 in HG002 from stLFR linked-reads by different lengths of flanking regions in RegionIndel.

## Data Availability

10x linked-reads library for HG002 used in this study is available at NCBI (https://www.ncbi.nlm.nih.gov/sra/SRX5608970) under accession code PRJNA527321. stLFR linked-reads library for HG002 is available at GiaB (https://ftp-trace.ncbi.nlm.nih.gov/ReferenceSamples/giab/data/AshkenazimTrio/HG002_NA24385_son/stLFR/). The VCF files used for the analyses are publicly accessible at https://zenodo.org/doi/10.5281/zenodo.13152036.

## Abbreviations

SVs: Structural Variants
SNPs: Single Nucleotide Polymorphism
Indels: Small insertions and deletions
WGS: Whole genome sequencing
PacBio: Pacific Biosciences
ONT: Oxford Nanopore Technologies
CCS: Circular consensus sequencing
stLFR: Single tube long fragment reads
GiaB: Genome in a Bottle
VCF: Variant call format
BAM: Binary Alignment Map

## Declarations

### Code availability and requirements

Project name: RegionIndel Project home page: https://github.com/maiziezhoulab/RegionIndel Operating system(s): Linux Programming language: Python Other requirements: Python 3 or higher. License: The MIT License.
