# Supplementary material for: Large indel detection in region-based phased diploid assemblies from linked-reads
Source: BMC Genomics. 2025 Mar 18;26(Suppl 2):263. doi: 10.1186/s12864-025-11398-z (PMC11916464; doi:10.1186/s12864-025-11398-z)
Supplement: Supplementary file 1 — Additional file 1. Evaluation of deletions ($\ge$≥50bp) on chromosome 21 in HG002 from stLFR linked-reads by different lengths of flanking regions in RegionIndel. [file 12864_2025_11398_MOESM1_ESM.pdf]

Large indel detection in region-based phased diploid assemblies from linked-reads

Can Luo, Brock A. Peters, and Xin Maizie Zhou

Supplementary Information

Deletions		flanking region (10kb)	flanking region (25kb)	flanking region (50kb)	flanking region (100kb)
50-1k	benchmark	58			
	TP	35	48	48	49
	TP_GT	28	40	41	39
	FP	28	53	76	123
	FN	23	10	10	9
	Recall	60.3%	82.8%	82.8%	84.5%
	Precision	55.6%	47.5%	38.7%	28.5%
	F1	57.9%	60.4%	52.7%	42.6%
	GT_accuracy	80.0%	83.3%	85.4%	79.6%
1k-10k	benchmark	7			
	TP	3	6	6	5
	TP_GT	3	6	6	5
	FP	0	1	7	6
	FN	4	1	1	2
	Recall	42.9%	85.7%	85.7%	71.4%
	Precision	100.0%	85.7%	46.2%	45.5%
	F1	60.0%	85.7%	60.0%	55.6%
	GT_accuracy	100.0%	100.0%	100.0%	100.0%

Table S1: Evaluation of deletions (>=50bp) on chromosome 21 in HG002 from stLFR linked-reads by different lengths of flanking regions in RegionIndel. Abbreviations: true positives (TP), false positives (FP), false negatives (FN), and genotype (GT).

Insertions		flanking region (10kb)	flanking region (25kb)	flanking region (50kb)	flanking region (100kb)
50-1k	benchmark	95			
	TP	14	19	23	22
	TP_GT	8	13	12	10
	FP	1	3	5	8
	FN	81	76	72	73
	Recall	14.7%	20.0%	24.2%	23.2%
	Precision	93.3%	86.4%	82.1%	73.3%
	F1	25.5%	32.5%	37.4%	35.2%
	GT_accuracy	57.1%	68.4%	52.2%	45.4%
1k-10k	benchmark	16			
	TP	1	1	1	1
	TP_GT	0	0	0	0
	FP	0	0	0	0
	FN	15	15	15	15
	Recall	6.2%	6.2%	6.2%	6.2%
	Precision	100.0%	100.0%	100.0%	100.0%
	F1	11.8%	11.8%	11.8%	11.8%
	GT_accuracy	0.0%	0.0%	0.0%	0.0%

Table S2: Evaluation of insertions (>=50bp) on chromosome 21 in HG002 from stLFR linked-reads by different lengths of flanking regions in RegionIndel. Abbreviations: true positives (TP), false positives (FP), false negatives (FN), and genotype (GT).

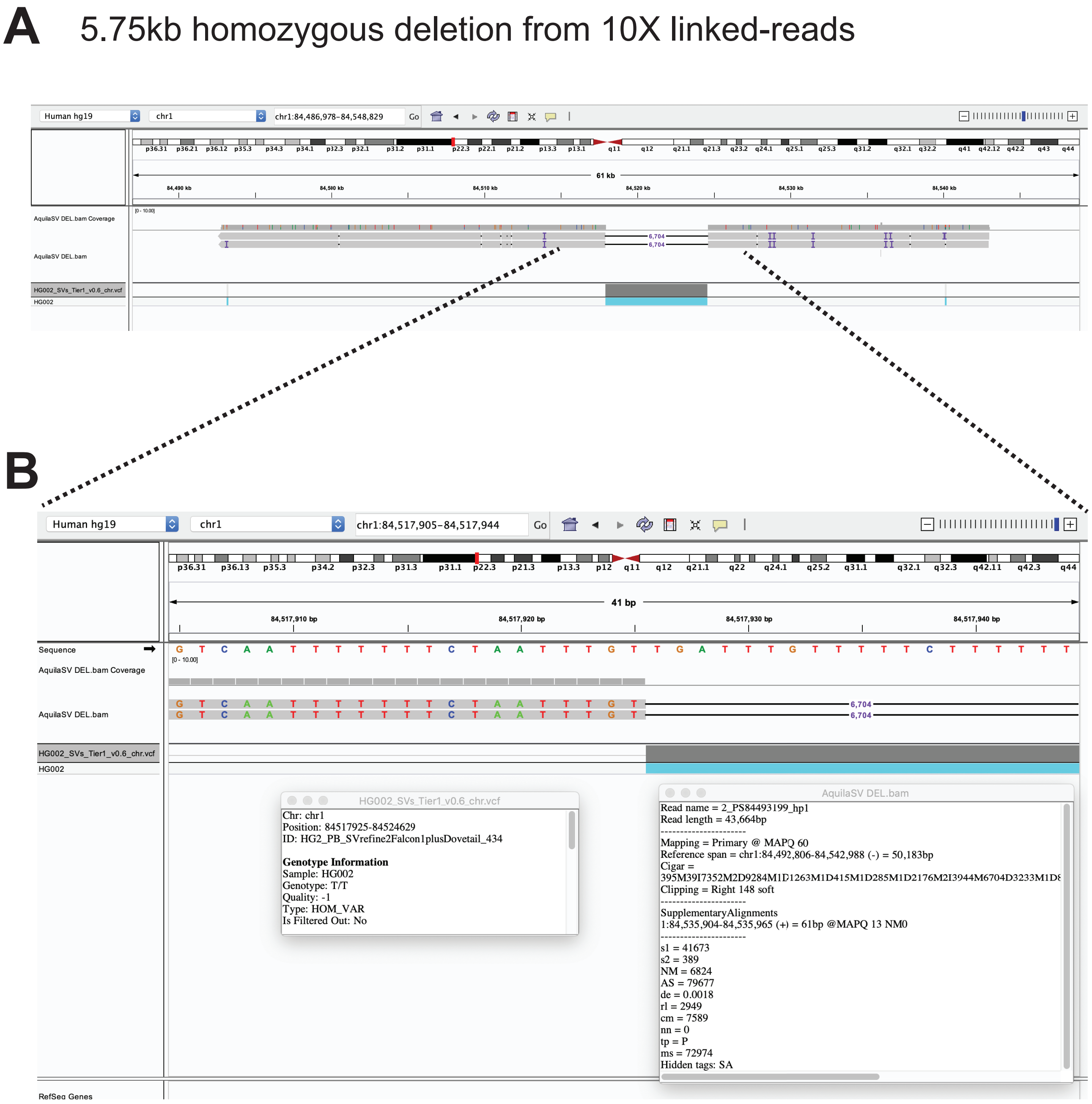

Fig. S1: One example of 5.75kb homozygous deletion from the 10X linked-reads data by RegionIndel. A. The IGV displays assembled diploid contigs from AquiaSV. It includes tracks for the contig BAM file from RegionIndel and the GiaB benchmark VCF file. The contigs also show there are several variants in the nearby region. B. It is a zoom-in view of the target SV in the center. The left inset box has the description about this benchmark SV, and the right inset box has the description about the contig information generated by RegionIndel. The gold standard supports this homozygous deletion. The contigs indicates this 6704bp deletion is identified by contigs from both haplotypes.

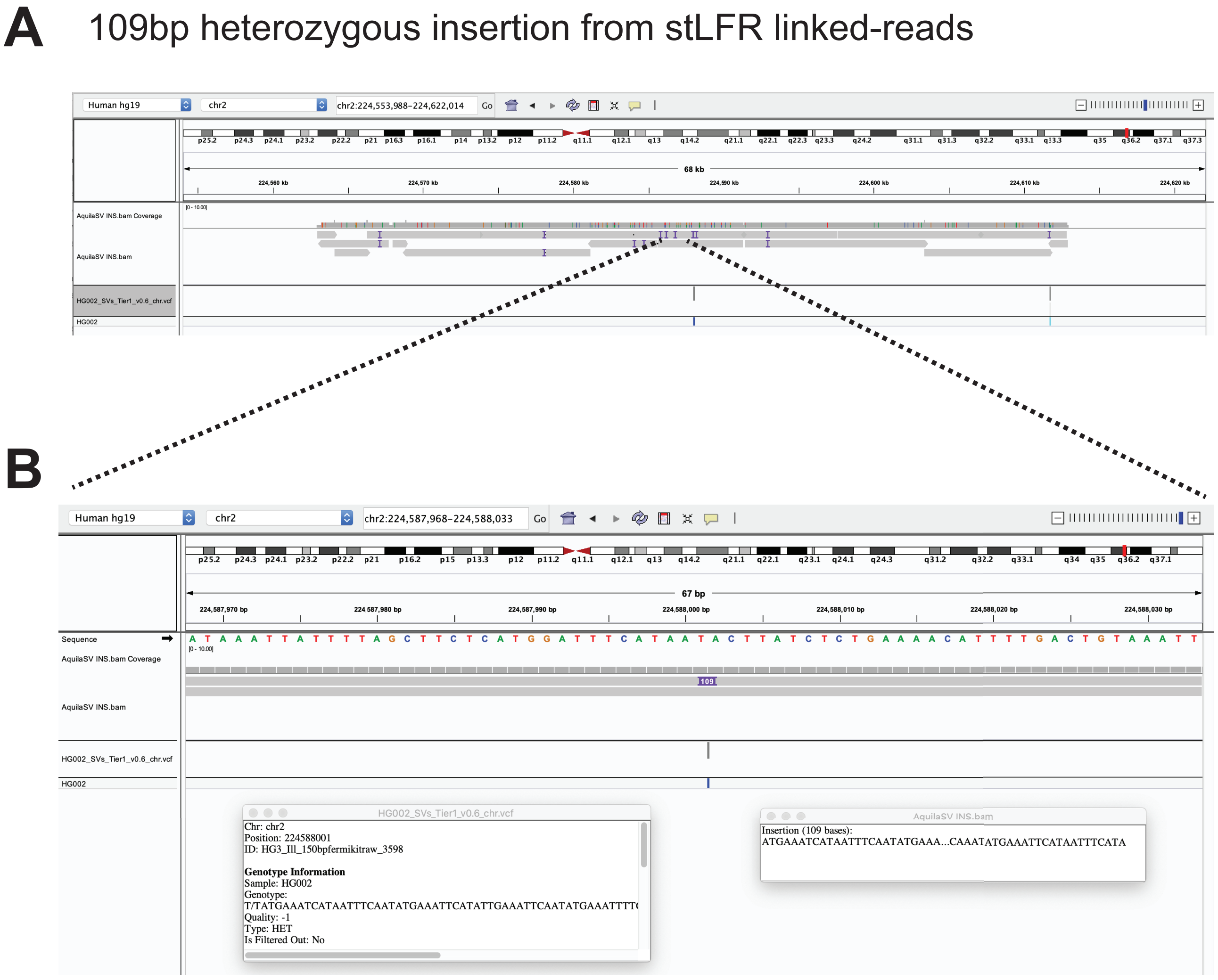

Fig. S2: One example of 109bp heterozygous insertion from the stLFR linked-reads data by RegionIndel. A. The IGV displays assembled diploid contigs from AquiaSV. It includes tracks for the contig BAM file from RegionIndel and the GiaB benchmark VCF file. The contigs also show that there are several variants in the nearby region. B. It is a zoom-in view of the target SV in the center. The left inset box has the description about this benchmark SV, and the right inset box has the description about the contig information generated by RegionIndel. It indicates that this insertion is identified from the contig of haplotype1. The gold standard supports this heterozygous insertion.
